# Assessment of Sleep, K-Complexes, and Sleep Spindles in a T21 Light-Dark Cycle

**DOI:** 10.3389/fnins.2020.551843

**Published:** 2020-10-06

**Authors:** Scott H. Deibel, Ryan Rota, Hendrik W. Steenland, Karim Ali, Bruce L. McNaughton, Masami Tatsuno, Robert J. McDonald

**Affiliations:** ^1^Department of Neuroscience, University of Lethbridge, Lethbridge, AB, Canada; ^2^NeuroTek Innovative Technology Inc., Toronto, ON, Canada; ^3^Department of Neurobiology and Behavior, University of California, Irvine, Irvine, CA, United States

**Keywords:** sleep, circadian rhythms, rats, memory, circadian misalignment

## Abstract

Circadian rhythm misalignment has a deleterious impact on the brain and the body. In rats, exposure to a 21-hour day length impairs hippocampal dependent memory. Sleep, and particularly K-complexes and sleep spindles in the cortex, have been hypothesized to be involved in memory consolidation. Altered K-complexes, sleep spindles, or interaction between the cortex and hippocampus could be a mechanism for the memory consolidation failure but has yet to be assessed in any circadian misalignment paradigm. In the current study, continuous local field potential recordings from five rats were used to assess the changes in aspects of behavior and sleep, including wheel running activity, quiet wakefulness, motionless sleep, slow wave sleep, REM sleep, K-complexes and sleep spindles, in rats exposed to six consecutive days of a T21 light-dark cycle (L9:D12). Except for a temporal redistribution of sleep and activity during the T21, there were no changes in period, or total amount for any aspect of sleep or activity. These data suggest that the memory impairment elicited from 6 days of T21 exposure is likely not due to changes in sleep architecture. It remains possible that hippocampal plasticity is affected by experiencing light when subjective circadian phase is calling for dark. However, if there is a reduction in hippocampal plasticity, changes in sleep appear not to be driving this effect.

## Introduction

Circadian rhythm disruption can have many harmful effects on the individual, such as cognitive impairment, higher mortality rate, metabolic syndrome, and higher incidences of cancer, dementia, and Alzheimer’s disease (Tranah et al., 2010, 2011; Escobar et al., 2011; Coogan et al., 2013; Baron and Reid, 2014; Zelinski et al., 2014a). As more and more people are being exposed to artificial light at night and the prevalence of shiftwork is increasing, the mechanisms underlying the various effects of circadian rhythm manipulations need to be understood (De Assis et al., 2003; Haus and Smolensky, 2006; Escobar et al., 2011). Animal models are crucial to uncovering the mechanisms for the deleterious effects elicited from these manipulations.

In rats, our lab studies the effect of light-dark cycles of a day length outside of the range of entrainment on learning and memory (Devan et al., 2001; Craig and McDonald, 2008; McDonald et al., 2013; Zelinski et al., 2013, 2014b; Deibel et al., 2014, 2019; Newman et al., 2019; Lewis et al., 2020). A light dark cycle in which the periodicity is anything other than 24 h is called a T cycle (Stephan, 1983; Campuzano, 1998). Our lab uses a T21, which means that from one cycle to the next, the lights are going on and off every 21 h. In other words, the period, or day length of the cycle is 21 h. When exposed to a T21, rats cannot adjust to the 21-hour day and instead oscillate according to their natural free-running endogenous period of approximately 24.5 h (tau) (Devan et al., 2001; Craig and McDonald, 2008; Zelinski et al., 2013, 2014b; Deibel et al., 2019). Amazingly impairments in the Morris water task can be evidenced with just 6 days of exposure (Devan et al., 2001; Zelinski et al., 2014b). Free-running in a light dark cycle means that the organisms internal time (circadian time) conflicts with the time dictated by the environment (zeitgeber time). So, processes that occur primarily in one phase of the light dark cycle, are now occurring more often in the contradictory phase. For example, rats are nocturnal and are thus mostly active during the dark. Because rats cannot synchronize to a 21-hour day, they continue to oscillate at roughly a 24-hour day, which means that significantly more activity now occurs when the lights are on. This is a type of circadian rhythm misalignment that occurs during shiftwork and jetlag (Baron and Reid, 2014). Other types of circadian misalignment, include misalignment between central and peripheral rhythms, or misalignment of eating from the sleep-wake cycle (Baron and Reid, 2014).

The failure to entrain in a light dark cycle as seen in our model occurs in nature with some individuals failing to entrain to a 24-hour day and instead free-running at approximately 25 h (Hayakawa et al., 2005). This is referred to as non-24-hour sleep-wake disorder (N24HSWD) and involves a continual state of circadian misalignment that varies in magnitude depending on when night time is occurring in relation to the phase of the endogenous clock (Hayakawa et al., 2005; Baron and Reid, 2014). The problem arises because one is forced to live according to a schedule that it cannot entrain to. In our model, when memory task training occurs during T21 exposure, similar to forced desynchrony models, we also advance the time of training (T23) to further promote misalignment (Devan et al., 2001; Deibel et al., 2019). This means that the circadian time of training changes with every passing day.

The mechanisms for the memory impairments observed in our and other models that manipulate circadian rhythms are largely unknown (Tapp and Holloway, 1981; Fekete et al., 1985, 1986; Ruby et al., 2008; Gibson et al., 2010; Loh et al., 2010, 2015; Fernandez et al., 2014; Lewis et al., 2020). As sleep is thought to be involved in memory consolidation (Diekelmann and Born, 2010; Logothetis et al., 2012), and is controlled in part by circadian mechanisms (Borbely and Tobler, 1985; Achermann and Borbely, 2003; Saper et al., 2005), its alteration has been discussed as a potential candidate (Zelinski et al., 2014a).

Sleep can be broken down into two broad categories: rapid eye movement (REM) and non-REM (NREM) sleep (Dement and Kleitman, 1957a,b; Dement, 1958; Jouvet, 1967). REM sleep consists of periods of rapid eye movements and twitching of the fingers and toes. Other than these movements, muscles are inactive and a sleeper remains relatively still. Using EEG or local field potentials (LFP) from the cortex, REM sleep is also characterized by high frequency and low amplitude cortical electrical activity. NREM sleep consists of periods between REM sleep. For humans they are divided into three stages (N1, N2, and N3) where the stage N3 is the deepest sleep (Iber, 2007). NREM sleep stage N3 is characterized by low frequency and high amplitude cortical electrical activity and it is also called slow wave sleep (SWS) (Diekelmann and Born, 2010). In rodents, because slow wave is a dominant brain activity for entire NREM sleep, rodent’s NREM sleep is often treated as a single sleep stage. Thus, the terms NREM sleep and SWS are used interchangeably. During neocortical slow wave oscillations in SWS, a large amplitude waveform (K-complex) can be observed, which is often followed by low amplitude oscillatory activity (sleep-spindles) (Crowley et al., 2002; Diekelmann and Born, 2010). In the hippocampus, strong theta oscillations are observed during REM sleep while slow oscillations with sharp wave ripples (SWRs) dominate NREM sleep. SWRs are characterized by high frequency LFP events that are likely to be generated by the synchronous firing of large populations of hippocampal CA3 pyramidal cells (Buzsáki, 1996; Jadhav and Frank, 2014). The reactivation (replay) of episodic memory primarily occurs during NREM sleep, (Wilson and McNaughton, 1994; Jadhav and Frank, 2014), specifically during SWRs (Kudrimoti et al., 1999). It has also been reported that the disruption of SWRs impairs acquisition of episodic memory (Buzsáki, 1996; Ego-Stengel and Wilson, 2010; Jadhav et al., 2012; Jadhav and Frank, 2014). Taken together, it has been hypothesized that consolidation of episodic memory is mediated by the coordinated neural activities during the hippocampal SWRs and cortical spindles in NREM sleep.

In mice and rats, phase shifting the light-dark cycle (Sei et al., 1992; Altimus et al., 2008; Castanon-Cervantes et al., 2010; Loh et al., 2010; Van Dycke et al., 2015), or restricting feeding to the light phase of the cycle (Loh et al., 2015) results in sleep occurring at phases when it normally does not (lights off), but generally does not change the total amount of sleep, or sleep stages (Sei et al., 1992; Castanon-Cervantes et al., 2010; Loh et al., 2010). Exposure to a T22 cycle can result in desynchrony of oscillations in body temperature and REM sleep from those in SWS and sleep-wake (Cambras et al., 2007). To our knowledge, no previous study has assessed sleep in rats exposed to a T21 light dark cycle. It remains a possibility that as with a T22, during T21 exposure there is misalignment among different oscillators involved in the sleep-wake cycle. Using continuous LFP recordings from five male Long Evans rats, we assess how aspects of sleep are affected by 6 days of T21 exposure that features 9 h of light and 12 h of dark (9L:12D) every cycle. In essence, the period of the light dark cycle was experimentally shortened by reducing the light phase by 3 h each day. We were interested in using just 6 days of T21 exposure because we observe memory impairments in male Long Evans rats when this manipulation occurs during training (Devan et al., 2001) or in between training and memory testing (Zelinski et al., 2014b). Finally, we also investigated aspects of sleep thought to be integral to hippocampal dependent memory, such as K-complexes, sleep spindles, and the cortical-hippocampal interaction (assessed in three of the five animals). The study aims to narrow the scope of potential candidate mechanisms for circadian manipulations that elicit memory impairments.

## Materials and Methods

### Animals

Two groups of eight male Long-Evans rats (average weight = 641.43 ± 22.634 g, 6-month-old) were surgically implanted with LFP electrodes (described below). After surgical implantation the rats were given one to 2 weeks of recovery time before being housed in the recording cages. The subjects had food and water available *ad libitum* during the recovery period and in the recording cages. Additionally, the rats had access to a running wheel in the recording cages. Six of the eight implanted rats were selected to be permanently placed into the recording housing units. Selection of these six rats was determined by quality and stability of LFP for a period of 3–4 weeks after the recovery period. The rats were handled daily prior to surgery and while housed in the recording cages prior to beginning the experimental recording. Once the recording had begun, contact with humans was minimized; apart from the scheduled cage cleaning with food and water replacement (Approximately 10 min at the fixed time on Monday, Wednesday, and Friday. Schedule was maintained during the T21 cycle). All procedures were conducted in accordance with the Canadian Council for Animal Care and approved by the University of Lethbridge Animal Welfare Committee.

### Animal Housing

Housing units were constructed to allow the rats to continuously live in a recording environment for the entire period of the experiment (Supplementary Figure S1). These units were designed to provide low noise continuous record for freely behaving rats. The housing units were made of an aluminum frame in which tempered glass (to reduce electrostatic interference) is inserted to create a 40 × 40 × 40 cm box. A bedding tray was placed at the bottom of the box and was easily removed horizontally for cleaning. The roof was made of aluminum and two large holes were cut for a tether cable and video monitoring. A running wheel with the diameter of 30.5 cm was fixed to the inside back wall of each housing unit. Water bottles and feed containers were fixed to the side of the housing units for ad-lib access.

### Data Acquisition

For the first group of six rats which were selected from 8 rats by quality of baseline recording, a first-generation Avatar system (Electrical Geodesics Inc., Eugene, OR, United States), which was developed for human EEG recordings, was adapted for rat use. This system was capable of recording at a 256 Hz sampling rate with up to six referenced channels. The recording signals were transmitted in real time to a host computer by Bluetooth with 16 bit sample size and a 6.5 mV dynamic range. The second group of six rats, again selected from eight rats by quality of baseline recording, were recorded on a second-generation Avatar 3000 (Electrical Geodesics Inc., Eugene, OR, United States) series. It allowed recording at a 2000 Hz sampling rate to an internal microSD card, which was replaced each week. It also had an average 250 Hz Bluetooth transmission rate that was used for monitoring LFP activity during continuous recording. The sampling size and dynamic range were expanded to 24 bits and 2.25 V, respectively. Once the recording was completed, the quality and stability of the hippocampal and cortical LFP signals were checked. These processes removed seven rats from twelve, leaving five animals for the analyses in this paper. We represent those animals as Rat1-1, Rat1-2, Rat2-1, Rat2-2, Rat2-3 where the first digit represents the batch of recordings (1 or 2) and the second digit represents the rat ID (1 to 3). For all analyses except the cortex-hippocampus interaction, a total of five animals were used, where three animals were used for the cortex-hippocampus interaction analysis because two animals had a recording malfunction or poor hippocampal LFP quality for several days: Animals used for the sleep analysis are Rat1-1, Rat1-2, Rat2-1, Rat2-2, Rat2-3; Animal used for the cortex-hippocampus interaction analysis are Rat1-1, Rat1-2, and Rat2-1.

The number of animals included in the final analyses decreased significantly from the initial number of 16. This was primarily due to the nature of the project in which the duration of recording was significantly longer than conventional electrophysiological recordings from freely behaving rodents, where recording duration is typically 2–3 h per day and the recording system is reconditioned every day. In this study, LFP recording was performed continuously over one month without stopping the system. Many unfavorable events such as scratching cables and hitting the head against walls happened during the continuous recording period because animals were allowed to move freely. Accumulation of these events decreased the quality of recording. However, despite the attrition, due to the within nature of the experiment these n’s are still considered adequate.

### Electrode and Surgical Protocol

Bipolar electrodes were constructed for LFP recording. The electrodes consisted of medical grade 40 AWG (Sigmund Cohn, MT Vernon, NY, United States, part #316SS3T) stainless steel wire coated in polytetrafluoroethylene (Teflon). Pre-surgery electrode impedances ranged from 50 to 100 K ohms. Two pairs of electrodes were implanted; the first twisted pair was implanted to the hippocampus (HC, −3.84 mm from Bregma, 2.4 mm lateral and 2.4 mm depth; tip separation of 0.4 mm). The second twisted pair was implanted to medial prefrontal cortex (mPFC, 2.76 mm from Bregma, 2.8 mm lateral and 4.25 mm depth at a 55 degree angle from dura; tip separation of 1.8 mm). In addition, two mono-electrodes (Cooner Wire, Chatsworth, CA, United States, part #AS 631) was sutured bilaterally to the neck (nuchal) muscle for EMG (electromyogram) recordings. A single stainless steel ground screw was also implanted in each rat’s cranium (−2 mm from Lambda). Depth and positions of electrodes were confirmed by histological analysis.

### T21

The room was maintained on a 12:12-h light:dark (2 and 320 lux, respectively) cycle, consisting of lights off at 8 am and on at 8 pm. For a period of 3–4 weeks after the surgery, wheel running activity, neck muscle EMG, and LFPs in HC and PFC were monitored for each group of eight rats to confirm stable recordings. Six of the eight rats were then selected to be placed in the permanent housing units. Once light cycle entrainment was confirmed using wheel running and EMG activity, the recording was divided into three stages; pre-T21, T21, and post-T21 stages (see Table 1). The days preceding the acute phase shift (days 1–6) constituted the pre-T21 exposure phase. The rats were then exposed to an asymmetrical T21 cycle with 43% light (L9:D12) (Craig and McDonald, 2008) for 6 days (days 7–12). The post-T21 stage consisted of 6 days of re-entrainment to 12:12-h light-dark cycle that was delayed by 3 h in comparison to the original pre-T21 light-dark cycle (days 13–19).

**TABLE 1:** Phase-advance schedule.

Days	Lights Off – Lights On
1–6	8:00–20:00
7	5:00–17:00
8	2:00–14:00
9	23:00–11:00
10	20:00–8:00
11	17:00–5:00
12	14:00–2:00
13–19	11:00–23:00

*Days 1–6 are the pre-entrainment stage with lights off at 8 am and on at 8 pm. Days 7–12 represent days in which lights off was phase-advanced 3 h per day. Days 13–19 are the post-entrainment stage with lights off at 11 am and on at 11 pm.*

### Offline Sleep Scoring and Data Analysis

Analysis has been performed on 18 days of continuous LFP recordings; 6 days of pre-T21 exposure (Pre), 6 days of T21 exposure (Shift), and 6 days of post-T21 exposure stage (Post). A spectrogram of the hippocampal LFP was calculated using a Hamming window of 2 s with no overlap. The EMG signal was filtered with a high-pass filter at 10 Hz to obtain movement-related muscle tone. Three researchers with expert knowledge on electrophysiological recordings were presented with this LFP spectrogram data and associated filtered EMG signal, and they manually scored the data for the brain states of motionless, quiet wake, REM sleep, and SWS. Video recording of animal behavior was also used when necessary. This was used to fine-tune the parameters for the automated scoring that was used to analyze the rest of the data.

**FIGURE 1:**
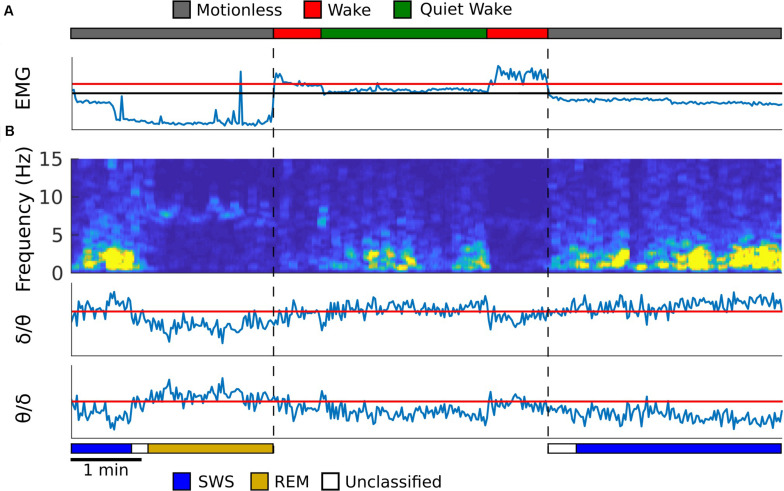
Automated sleep state detection. **(A)** EMG from the neck muscle (blue trace) was used to score periods of motionless, wake and quiet wake, which were represented as gray, red and green periods in the top trace. EMG power is shown, and the *y*-axis is in log scale. Red threshold was used to find periods of low EMG power, and the black threshold was used to separate quiet wake from motionless periods. **(B)** A spectrogram was created from the hippocampal LFP (first panel). The average spectral power for delta (1–4 Hz) and theta (5–10 Hz) frequency bands was calculated and the theta to delta ratio (second) and the delta to theta ratio (third) were computed. Within motionless periods, these two ratios were used to identify periods of REM (dark yellow) and SWS (blue), respectively. The periods that did not exceed the thresholds was labeled as unclassified (white). All thresholds were determined so that the agreement between manual scoring and automated scoring was maximized. Temporal resolution for each plot is 2 s.

Refer to Figure 1 for an example from one rat, and Supplementary Figure S2 for examples from the remaining rats, of how the algorithm detected sleep states. The first step for the automated scoring algorithm is to identify behavioral states (wake, quiet wake, and motionless) using EMG power, which is computed by averaging the EMG spectrogram values for frequencies 10 Hz and above. Two thresholds were set to find these states. One threshold was set to find periods of low EMG power that separates quiet wake and motionless from wake. A second threshold was to separate periods of quiet wake and motionless within the periods of low EMG power. The periods above the second threshold were classified as quiet wake and the ones below were considered to be periods of motionless (Figure 1A). The thresholds were adjusted so that the agreement between manual scoring and automated scoring is maximized by using Bayesian optimization (Mockus et al., 1978).

The second step is to detect REM sleep and SWS in motionless episodes using the spectrogram of the hippocampal LFP signal. Because SWS and REM sleep are characterized by strong delta oscillation (1–4 Hz) and theta oscillation (5–10 Hz), respectively, mean delta power and theta power were calculated from the hippocampal spectrogram. A ratio of theta to delta power was used to find REM sleep epochs, and a ratio of delta to theta power was used to find slow wave sleep epochs (Louie and Wilson, 2001; Figure 1B). A threshold for each of these ratios was determined by comparing the resulting epochs against manually scored spectrogram data for each animal. If neither the SWS or REM thresholds were met with their respective power ratios then that period of time was left unclassified (Figure 1B). See Supplementary Figure S3A for the agreement between manual scoring and the computer algorithm.

**FIGURE 2:**
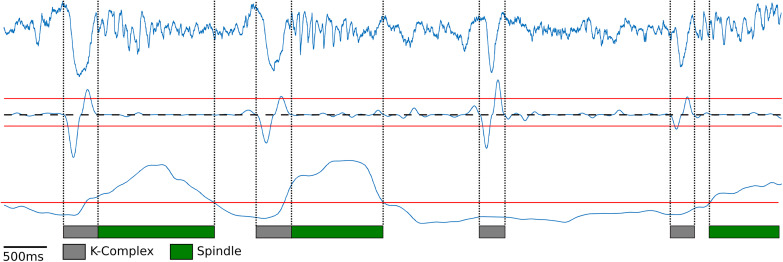
K-Complex and spindle detection. Raw cortical local field potentials (top blue trace) recorded at 250 Hz showing representative K-Complexes and Spindles. K-Complex slope (middle blue trace) was computed by filtering the cortical EEG between 0.75 and 6 Hz and computing the difference between adjacent points to determine the slope. Peaks above the upper threshold (red line) and below the lower threshold (red line) were found. Lower peaks were matched to upper peaks within one second to find candidate K-complexes. Edges were then expanded until the slope crosses zero (dashed black line). Spindle power (bottom blue trace) was computed by filtering between 10 and 20 Hz and finding periods exceeding the manually selected threshold (bottom red line).

Next, K-complexes were detected during slow wave sleep. Similar to the humans’ case, K-complexes in rodents are characterized by a large down-ward fluctuation in the cortical LFP trace caused by the simultaneous silencing of neurons during a down state event (Johnson et al., 2010; Mak-McCully et al., 2014; Figure 2, top blue trace). K-complexes in both humans and rodents are often followed by spindles, suggesting that they may play a role in memory consolidation during sleep (Tononi and Cirelli, 2006). To detect K-complexes as a large down-ward fluctuation within the cortical LFP trace, first, the cortical LFP trace was bandpass filtered using a Chebyshev Type I filter between 0.75 and 6 Hz during motionless episodes. The instantaneous slope (derivative) was computed by calculating the difference between adjacent signal values in the filtered LFP trace (Figure 2, middle blue trace). Using this slope two thresholds were determined: the 98th percentile of the negative slope values and the 95th percentile of the positive slope values for the downward and upward slope thresholds, respectively (Figure 2, red lines above and below the middle blue trace). These thresholds were determined using information from the manually scored data: For each rat 1 h of cortical LFP data was manually scored for K-complexes and the thresholds were set to achieve maximal agreement (Supplementary Figure S3B). Local minima slope values less than the negative slope threshold were used to mark potential start of K-complex times and local maxima values greater than the positive slope threshold were used to mark potential end times. Initial detection of K-complexes was performed by matching start and end times which occur within one second of each other. Start times were then moved backward to the first time point where the calculated slope value has a value greater than or equal to zero (Figure 2, dashed black line) to ensure the entire length of the K-complex event is contained within the timestamps. A similar procedure was performed for end time points as well, finding the first value less than or equal to zero (Figure 2, dashed black line). K-complexes were required to have a minimum distance of 200 ms separating the local minima (Figure 2, gray bars represent detected K-complexes).

In order to detect sleep spindles, the cortical LFP trace during motionless periods was bandpass filtered between 10 and 20 Hz, following previous rodent literature (Kandel and Buzséki, 1997; Johnson et al., 2010; Eckert et al., 2020). The range of characteristic frequency for rodent’s spindles is slightly different from that for humans where they are defined as bursts of 10–15 Hz activity (Purcell et al., 2017). However, recent studies revealed that LFP recordings from rodents’ deep cortical layers, which is the case in this experiment, exhibit a comparable profile of spindles in humans (Andrillon et al., 2011; Peyrache et al., 2011; Astori et al., 2013). Spindle power was calculated by rectifying the filtered signal by squaring the values (Figure 2, bottom blue trace). Spindle power exceeding a manually set threshold (Figure 2, bottom red line) was used to find spindles (Figure 2, green bars represent detected spindles). Like the K-complex detection, spindles were manually scored using 1 h of data for each rat, and the threshold that achieved the maximal agreement was used (Supplementary Figure S3C). In addition, to ensure that the spindles had at least two oscillations and that any momentary loss in spindle power did not count the same spindle multiple times, a minimum duration of spindles (200 ms) and minimum gap between spindles (700 ms) were imposed.

For each of the brain states described above a threshold needs to be set for the algorithm to work. In order to objectively find the optimal threshold value Bayesian optimization was used (Mockus et al., 1978). A threshold was initially set randomly for each of the sleep features. For each 2 second window used in the spectrogram the sleep state in that window was determined by the algorithm using this threshold. The number of windows where the sleep state found using the automated algorithm did not match the state found by manual scoring was counted. The thresholds were then adjusted in the direction that decreases this count. The set of thresholds that minimized this count for each of the sleep features was used. The procedure was run for 1000 iterations and was performed for each day and animal.

### Statistical Analysis

All circadian rhythm analyses were performed using ClockLab (Actimetrics, Wilmette, IL, United States). 6 min bins were used for all analyses and dependent variables. For all sleep circadian analyses except for K-complexes and sleep spindles, the percentage of time occupied per 6 min bin was used (Cambras et al., 2007). For K-complexes and sleep spindles the number of occurrences was used. For the sleep analyses, average amount per hour refers to the average amount of time in minutes spent per hour in that aspect of sleep for each stage of the experiment. For activity, K-complexes, and sleep spindles the average amount per hour refers to the average number of revolutions or occurrences per hour for each stage of the experiment. Due to a technical malfunction, Rat 2–3 (see Supplementary Figure S3) was missing one day of data across all of the sleep measures from the pre stage of the experiment. In this case the missing day was omitted from the analyses and thus the average for the pre stage of the experiment consisted of the remaining 5 days. The Chi squared periodogram was used to calculate the period (time it takes to complete one full oscillation of a rhythm) of wheel running activity and the aspects of sleep for the different stages of the experiment. Periods were tested at an alpha of 0.05 in the circadian range of 20–28 h. Animals that did not have a statistically significant period were excluded from the period analyses for that specific variable. This only occurred for quiet wakefulness, with one occurrence in the Pre stage of the experiment and two occurrences in the T21 stage of the experiment. Scale actograms for activity and “actogram like” figures for sleep and its associated components are double plotted.

To assess the cortex-hippocampus interaction, a triggered average in the hippocampal LFP was created for every K-complex. The amplitude between the peak and trough of the hippocampal LFP within ± one second from a K-complex, as well as the timing of this peak in relation to a k-complex were used as dependent measures. We also calculated the peak to trough width and the area under the main peak.

Finally, it is possible that T21 exposure could affect the transitions between sleep states. We counted the number of SWS to REM, REM to SWS, and total (summation of the two) transitions per hour during the three stages of the experiment.

In all cases, the data for each stage of the experiment were averaged and then compared with Friedman’s ANOVA. Dunn’s multiple comparisons were conducted when Friedman’s ANOVA was significant.

## Results

All analyses have an N of five, except for the cortex-hippocampus interaction analyses, which have an N of three. For each of the circadian outputs we provide average waveforms for all rats during the Pre (A), Shift (B,D), and Post (C) stages of the experiment. These waveforms provide the average temporal distribution of the process of interest per day of a given experimental stage. In the case of processes for which we measured the number of occurrences (activity, K-complexes, and Sleep spindles) the average number of counts per min for each 6-minute bin is plotted. In the case of all other aspects of sleep, the average percentage of time per minute for each 6-minute bin is plotted. For the shift stage of the experiment we provide two waveforms with different parameters. The first waveform (B) is folded with a 24-hour period triggered from the previous 12:12 LD cycle. The second waveform is folded with a 21-hour period and includes the new light and dark times during T21 exposure. T21 entrainment would be evidenced by arrhythmicity in B, and the appearance of a clear rhythm in D. Conversely, failure to entrain to the T21 would be evidenced by a pattern of activity in B similar to that during the pre stage of the experiment. This would be accompanied by the failure of a clear rhythm in D. To provide insight at the level of each individual animal, actograms of each rat for all of the circadian output variables are included in Supplementary Figure S3. For all subsequent sleep analyses, refer to Table 2 for means, standard errors of the mean, and significant *p* values.

**TABLE 2:** Circadian rhythm dependent measures for activity and sleep.

Activity	Measure	Pre		Shift		Post		Significance
	Dark Phase %	91.910	2.163	59.642	3.869	83.955	4.808	Pre > S 0.005
	Revolutions per Hour	29.418	6.403	23.829	4.737	26.882	5.400	NS
	Period (h)	23.76	0.175	24.24	0.216	23.82	0.111	NS
**QW**	Dark Phase %	45.296	1.988	56.219	1.653	46.477	0.983	NS
	Amount per Hour (min)	12.473	0.763	11.380	1.135	13.157	0.932	NS
	Period (h)	24.233	0.769	24.633	0.338	23.900	0.265	NS
**Motionless**	Dark Phase %	29.967	1.389	58.925	1.147	35.988	1.290	Pre < S 0.005
	Amount per Hour (min)	17.767	1.759	17.496	0.830	17.878	0.498	NS
	Period (h)	23.84	0.068	24.120	0.183	23.940	0.117	NS
**SWS**	Dark Phase %	29.684	1.357	59.159	1.186	36.099	1.402	Pre < S 0.005
	Amount per Hour (min)	11.265	0.694	10.987	0.439	11.865	0.573	NS
	Period (h)	23.880	0.086	24.100	0.192	24.180	0.256	NS
**REM**	Dark Phase %	30.926	1.642	57.771	1.699	35.312	1.434	Pre < S 0.005
	Amount per Hour (min)	4.192	0.664	3.927	0.325	3.681	0.275	NS
	Period (h)	25.160	0.728	24.36	0.465	24.040	0.112	NS
**K-complexes**	Dark Phase %	39.427	1.524	58.330	0.859	45.393	1.486	Pre < S 0.005
	Number per Hour	586.704	7.693	583.690	11.131	579.255	17.500	NS
	Period (h)	23.800	0.563	24.100	0.207	24.000	0.055	NS
**Sleep Spindles**	Dark Phase %	37.378	0.937	58.054	0.995	42.897	0.713	Pre < S 0.005
	Number per Hour	329.625	13.869	316.216	20.984	334.855	21.168	NS
	Period (h)	23.880	0.049	24.120	0.188	23.980	0.066	NS

*The first column for each stage of the experiment is the mean and the second column is the standard error of the mean. In the significance column, significant multiple comparisons and the associated *p* values are provided. For example, consider Activity Dark Phase % in the first row. As the Friedman’s ANOVA was significant, multiple comparisons were conducted. Pre was greater than Shift (S) with a *p* value of *p* = 0.005.*

### Wheel Running

**FIGURE 3:**
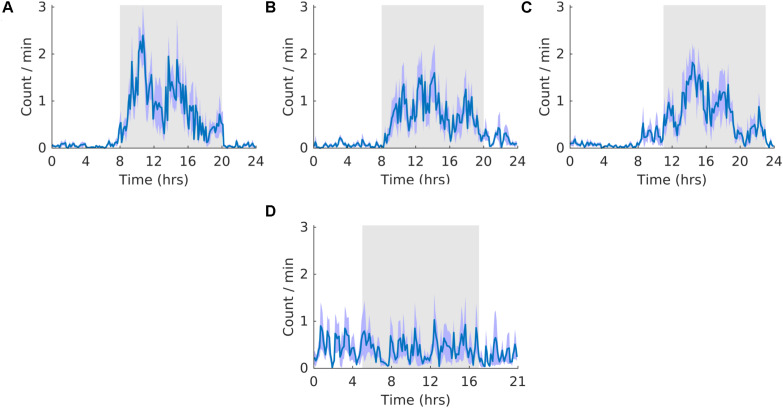
Running wheel activity. **(A)** Group average normalized waveforms for the six day pre-T21 stage of the experiment. **(B)** Group average normalized waveforms for the 6 days T21 stage of the experiment. **(C)** Group average normalized waveforms for the 6 day post-T21 stage of the experiment. **(D)** Group average normalized waveforms for the 6 day T21 stage of the experiment framed for a period of 21 h. The gray shaded area represents lights off, and the purple shaded area represents standard error of the mean.

Activity did not entrain to the T21 as the waveform folded with a 24-hour period during the shift stage of the experiment (Figure 3B) exhibited a pattern similar to that during the pre stage of the experiment (Figure 3A). This observation was also supported by no clear pattern in the waveform folded with a 21-hour period during the shift stage of the experiment (Figure 3D). Accordingly, the waveform during the post stage was entrained to a 24-hour period (Figure 3C). Fitting with a failure to entrain to the T21, there was less dark activity during T21 exposure [χ^2^(2) = 14, *p* = 0.001; *p* = 0.005] (Table 2, Activity). There was no change in period, or the average amount of activity per hour across the three experimental stages (Table 2, Activity). These data suggest that the T21 did not affect the amount of activity and was not entrainable with the animals maintaining a period of approximately 24 h during T21 exposure. Actograms of wheel running of each rat are found in the Supplementary Figure S4A.

### Quiet Wakefulness

**FIGURE 4:**
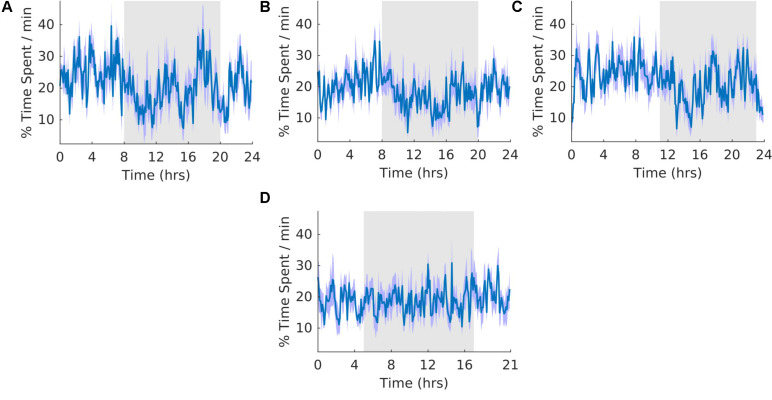
Quiet wakefulness. **(A)** Group average normalized waveforms for the 6 days pre-T21 stage of the experiment. **(B)** Group average normalized waveforms for the 6 days T21 stage of the experiment. **(C)** Group average normalized waveforms for the 6 day post-T21 stage of the experiment. **(D)** Group average normalized waveforms for the 6 days T21 stage of the experiment framed for a period of 21 h. The gray shaded area represents lights off, and the purple shaded area represents standard error of the mean.

The overall shape of the waveform did not change from pre, shift to post stages of the experiment (Figures 4A–C) and no clear pattern was observed in the waveform that was folded with a 21-hour period (Figure 4D), indicating that the T21 did not have an effect on quiet wakefulness. This was also supported by no statistically significant changes in the percentage of activity during the dark phase of the cycle, amount per hour, and period (Table 2, QW). Quiet wakefulness rhythmicity was more precarious, however, as one animal during pre-shift and two animals during the T21 did not have statistically significant periods. These data suggest that QW did not entrain to the T21, nor was its amount affected. Actograms of quiet wakefulness of each rat are found in the Supplementary Figure S4B.

### Motionless Sleep

**FIGURE 5:**
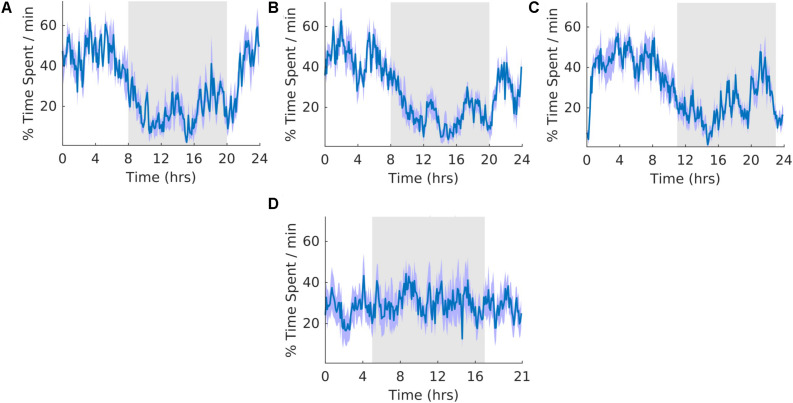
Motionless sleep. **(A)** Group average normalized waveforms for the 6 days pre-T21 stage of the experiment. **(B)** Group average normalized waveforms for the 6 days T21 stage of the experiment. **(C)** Group average normalized waveforms for the 6 days post-T21 stage of the experiment. **(D)** Group average normalized waveforms for the 6 days T21 stage of the experiment framed for a period of 21 h. The gray shaded area represents lights off, and the purple shaded area represents standard error of the mean.

The overall shape of the waveform did not change from pre, shift to post stages of the experiment (Figures 5A–C) and no clear pattern was observed in the waveform that was folded with a 21-hour period (Figure 5D), indicating that the T21 did not have an effect on motionless sleep. Fitting with a failure to entrain to the T21, there was more dark motionless sleep during T21 exposure [χ^2^(2) = 8.400, *p* = 0.009; *p* = 0.009] (Table 2, Motionless). There was no change in period, or the average amount of motionless sleep per hour across the three experimental stages (Table 2, Motionless). These data suggest that the T21 did not affect the amount of motionless sleep and was not entrainable with the animals maintaining a period of approximately 24 h during T21 exposure. Actograms of motionless sleep of each rat are found in the Supplementary Figure S4C.

### Slow Wave Sleep

**FIGURE 6:**
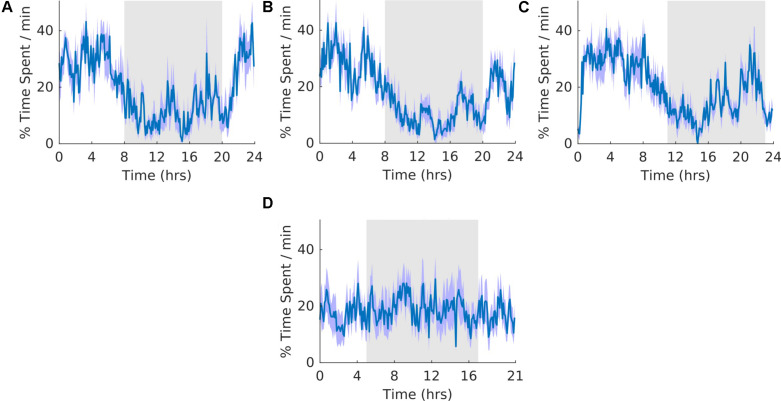
Slow wave sleep. **(A)** Group average normalized waveforms for the 6 days pre-T21 stage of the experiment. **(B)** Group average normalized waveforms for the 6 days T21 stage of the experiment. **(C)** Group average normalized waveforms for the 6 days post-T21 stage of the experiment. **(D)** Group average normalized waveforms for the 6 days T21 stage of the experiment framed for a period of 21 h. The gray shaded area represents lights off, and the purple shaded area represents standard error of the mean.

In our experiment, approximately 62–66% of motionless period was classified as SWS (Table 2, motionless and SWS). As a result, the general shape of waveforms for motionless and SWS was similar (Figures 5, 6). The overall shape of the waveform did not change from pre, shift to post stages of the experiment (Figures 6A–C) and no clear pattern was observed in the waveform that was folded with a 21-hour period (Figure 6D), indicating that the T21 did not have an effect on SWS. Fitting with a failure to entrain to the T21, there was more dark SWS during T21 exposure [χ^2^(2) = 10.000, *p* = 0.001; *p* = 0.005] (Table 2, SWS). There was no change in period, or the average amount of activity per hour across the three experimental stages (Table 2, SWS). These results confirmed that the T21 did not affect the amount of SWS and was not entrainable with the animals maintaining a period of approximately 24 h during T21 exposure. Actograms of SWS of each rat are found in the Supplementary Figure S4D.

### REM Sleep

**FIGURE 7:**
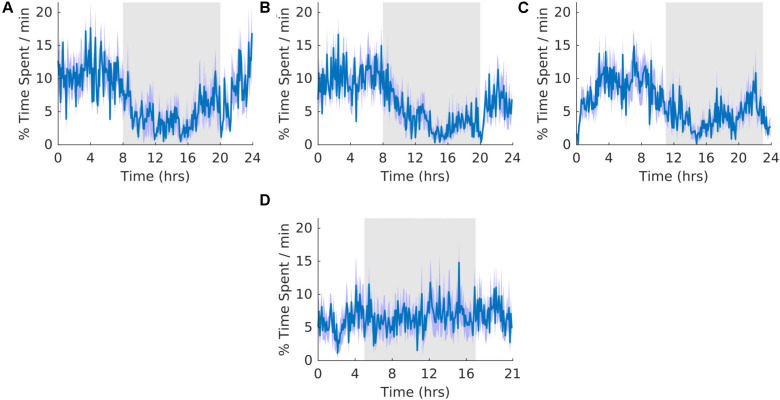
Rapid eye movement sleep. **(A)** Group average normalized waveforms for the 6 days pre-T21 stage of the experiment. **(B)** Group average normalized waveforms for the 6 days T21 stage of the experiment. **(C)** Group average normalized waveforms for the 6 days post-T21 stage of the experiment. **(D)** Group average normalized waveforms for the 6 days T21 stage of the experiment framed for a period of 21 h. The gray shaded area represents lights off, and the purple shaded area represents standard error of the mean.

The overall shape of the waveform did not change from pre, shift to post stages of the experiment (Figures 7A–C) and no clear pattern was observed in the waveform that was folded with a 21-hour period (Figure 7D), indicating that the T21 did not have an effect on REM sleep. Fitting with a failure to entrain to the T21, REM sleep did not entrain to the T21. In line with this, there was more dark REM sleep during T21 exposure [χ^2^(2) = 10.000, *p* = 0.001; *p* = 0.005] (Table 2, REM). There was no change in period, or the average amount of REM sleep per hour across the three experimental stages (Table 2, REM). These data suggest that the T21 did not affect the amount of REM sleep and was not entranable. Actograms of REM of each rat are found in the Supplementary Figure S4E.

### K-Complexes

**FIGURE 8:**
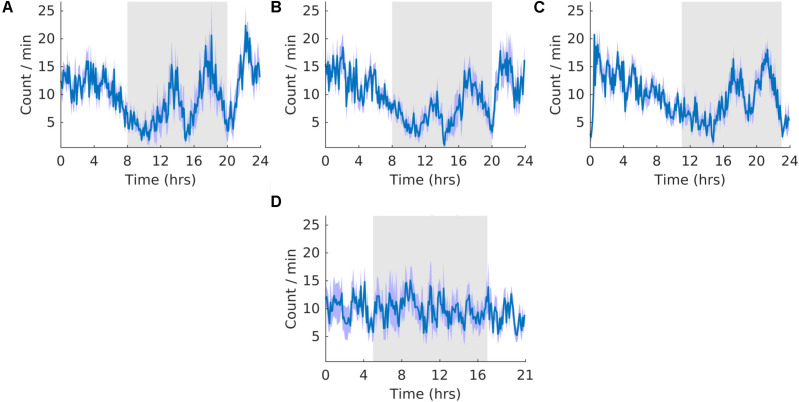
K-complexes. **(A)** Group average normalized waveforms for the 6 days pre-T21 stage of the experiment. **(B)** Group average normalized waveforms for the 6 days T21 stage of the experiment. **(C)** Group average normalized waveforms for the 6 days post-T21 stage of the experiment. **(D)** Group average normalized waveforms for the 6 days T21 stage of the experiment framed for a period of 21 h. The gray shaded area represents lights off, and the purple shaded area represents standard error of the mean.

Throughout the three stages, the count of K-complexes was highest at the beginning of the light cycle and decreased to the onset of dark cycle (Figure 8). However, there were also many K-complexes during the dark phase of the cycle, particularly toward the end (Figure 8). Qualitatively, as expected, these patterns of K-complexes were positively correlated with those of SWS and motionless and negatively correlated with that of wheel running during the dark phase. The overall shape of the waveform did not change from pre, shift to post stages of the experiment (Figures 8A–C) and no clear pattern was observed in the waveform that was folded with a 21-hour period (Figure 8D), indicating that the T21 did not have an effect on K-complexes. Fitting with a failure to entrain to the T21, there were more dark K-complexes during T21 exposure [χ^2^(2) = 10.000, *p* = 0.001; *p* = 0.005], and there was no change in period, or the average amount of K-complexes per hour across the three experimental stages (Table 2, K-complexes). These data suggest that the T21 did not affect the amount of K-complexes, and was not entrainable. Actograms of K-complexes of each rat are found in the Supplementary Figure S4F.

### Sleep Spindles

**FIGURE 9:**
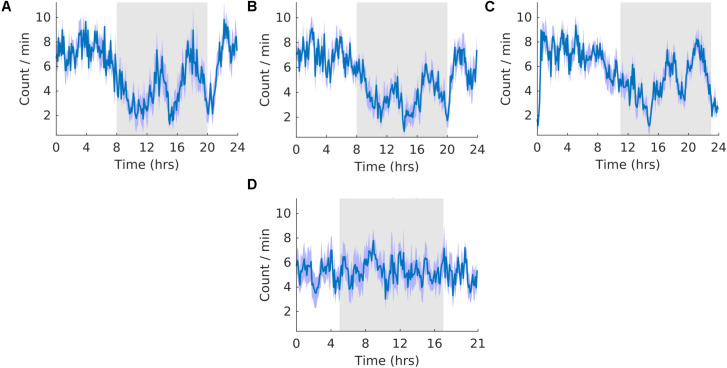
Sleep spindles. **(A)** Group average normalized waveforms for the 6 days pre-T21 stage of the experiment. **(B)** Group average normalized waveforms for the 6 days T21 stage of the experiment. **(C)** Group average normalized waveforms for the 6 days post-T21 stage of the experiment. **(D)** Group average normalized waveforms for the 6 days T21 stage of the experiment framed for a period of 21 h. The gray shaded area represents lights off, and the purple shaded area represents standard error of the mean.

The waveforms were highly similar to those of K-complexes because sleep spindles were often associated with K-complexes. The overall shape of the waveform did not change from pre, shift to post stages of the experiment (Figures 9A–C) and no clear pattern was observed in the waveform that was folded with a 21-hour period (Figure 9D), indicating that the T21 did not have an effect on sleep spindles. Fitting with a failure to entrain to the T21, There were more dark sleep spindles during T21 exposure [χ^2^(2) = 10.000, *p* = 0.001; *p* = 0.005] (Table 2, sleep spindles). There was no change in period, or the average amount of sleep spindles per hour across the three experimental stages (Table 2, sleep spindles). These results confirmed that the T21 did not affect the amount of sleep spindles and was not entrainable. Actograms of sleep spindles of each rat are found in the Supplementary Figure S4G.

### Sleep State Transitions

**FIGURE 10:**
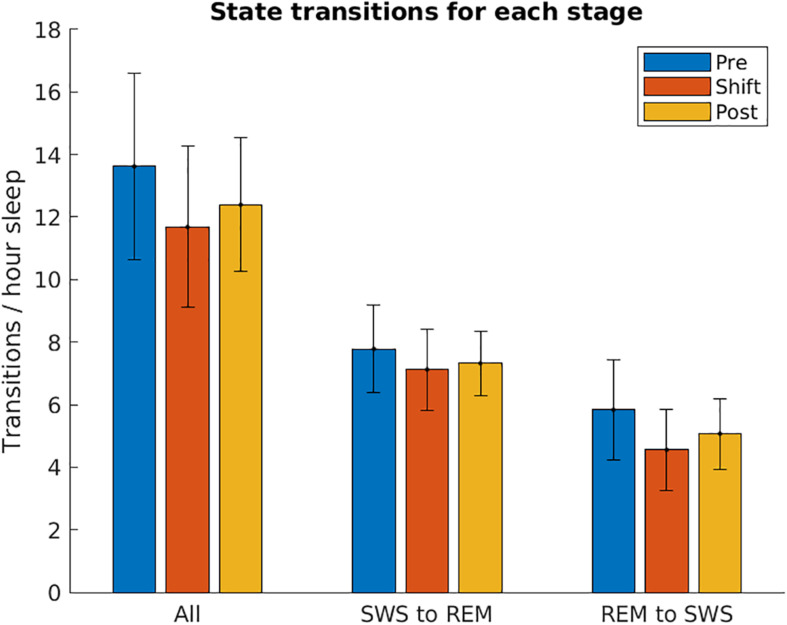
Sleep state transitions. The number of transitions from slow-wave sleep to REM sleep, and REM sleep to slow-wave sleep are shown for each stage of the experiment, normalized by the number of hours spent in sleep. These are averaged over the five animals used in the experiment. These two values were then summed together to show All transitions. Error bars represent SEM.

To investigate whether T21 exposure affects the transitions between SWS and REM, the number of sleep state changes per hour was counted across the different stages of the experiment. As demonstrated in Figure 10, there was no significant difference in the total [χ^2^(2) = 1.200, *p* = 0.691], SWS to REM [χ^2^(2) = 0.000, *p* > 0.999], or REM to SWS transitions [χ^2^(2) = 1.200, *p* = 0.691].

### Cortex-Hippocampus Interaction

**FIGURE 11:**
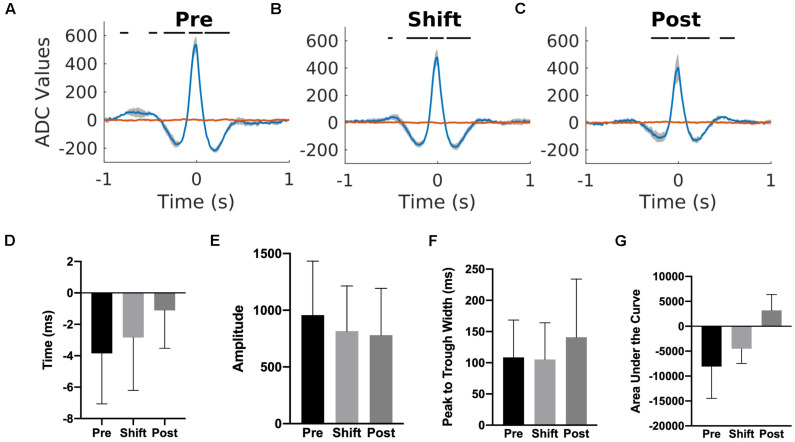
Cortex-hippocampus interaction during T21 exposure. **(A)** K-complex triggered hippocampal LFP during the pre-shift stage of the experiment. **(B)** K-complex triggered hippocampal LFP during the shift stage of the experiment. **(C)** K-complex triggered hippocampal LFP during the post-shift stage of the experiment. The red trace in each figure corresponds to the hippocampal LFP triggered by random time point during slow wave sleep. **(D)** The average timing of the peak in relation to a K-complex during the three stages of the experiment. Zero represents the center of a K-complex. **(E)** The average amplitude of the peak during the three stages of the experiment. **(F)** The average width of the peak during the three stages of the experiment. **(G)** The average area of the peak during the three stages of the experiment.

To investigate if a temporal relationship between the cortical and hippocampal activity was affected by the T21 cycle, K-complex triggered averages of the hippocampal LFP were computed for three animals. We used the K-complex because it was the cortical event that was detected more reliably than sleep spindles and its center was easily estimated due to the relatively simple shape of the K-complex. As depicted for one representative animal in Figures 11A–C, there was a peak in the K-complex triggered hippocampal LFP very shortly before the estimated center of a K complex (blue trace). If the average hippocampal LFP was calculated using random time points, the peak in the LFP disappeared (red trace). These results suggest that K-complexes and hippocampal LFPs were corelated. The correlation was not due to volume conduction because both cortical and hippocampal LFPs were collected by differential recordings between the tips in bipolar electrodes. To further confirm that it is not volume conduction, we also analyzed independent data sets with differential recordings from rat’s primary motor cortex (bipolar electrode tip distance of 1.8 mm) and hippocampus (bipolar electrode tip distance of 0.5 mm). We confirmed that a similar peak appeared in the K-complex triggered average hippocampal LFP, but this disappeared when random time points were used as the source of reference. T21 exposure had no effect on the timing of the peak in relation to K-complex center [χ^2^(2) = 4.000, *p* = 0.1667; Figure 11D], amplitude of the peak [χ^2^(2) = 4.667, *p* = 0.194; Figure 11E], width of the peak [χ^2^(2) = 3.445, *p* = 0.278; Figure 11F] and area under the curve [χ^2^(2) = 2.00, *p* = 0.528; Figure 11G].

### Summary

The present study assessed the effects of six consecutive days of exposure to a T21 cycle (L9:D12) on wheel running activity, quiet wakefulness, various aspects of sleep (motionless sleep, slow wave sleep, REM sleep, K-complexes, sleep spindles) and the cortex-hippocampus interaction during SWS in rats. The motivation was to determine if there were any changes in sleep that might account for the observed impairment in hippocampal dependent tasks that is elicited from a very similar T21 paradigm (Devan et al., 2001; Zelinski et al., 2014b). The amount of quiet wakefulness and sleep architecture was not affected by the T21. The persistence of a period that is approximately 24 h during T21 exposure in all circadian variables measured indicates that the schedule was outside the range of entrainment. This is further evidenced by less wheel running activity and more sleep-related activity in the dark phase of the photoperiods during the T21 (dark phase %). There was also no change in sleep state transitions across the three stages of the experiment. Finally, the cortex-hippocampus interaction during SWS as assessed by the K-complex triggered hippocampal LFPs was also unaffected during T21 exposure. In summary, the only aspect of sleep and activity that was affected was the temporal distribution of these processes. So, during T21 these processes were occurring at times in which they are usually inhibited.

### T-Cycles and Circadian Rhythms

The period of all of the circadian influenced outputs measured did not change during or after T21 exposure. A period of approximately 24 h during T21 exposure suggests that the current cycle was outside the range of entrainment. It should be noted, however, that we did not find an increase in period during T21 exposure that is characteristic of free-running rats. With the exception of REM sleep, period was increased in all other aspects of sleep and activity, although these effects were not statistically significant. It is likely that with more animals and days of exposure these output measures would have had an increased period during the T21.

T-cycles with various photoperiods can produce a strong free-running component and a weaker light-entrained component that could be an artifact of the light dark cycle rather than evidence of entrainment (Campuzano, 1998; Cambras et al., 2004, 2007; Vivanco et al., 2010; Casiraghi et al., 2012). In what the authors call an animal model of human internal desynchrony, various rhythms, including activity, sleep, and core-body temperature showed differential entrainment to a T22 cycle in rats (Cambras et al., 2007). The authors concluded that similar to what occurs in many human manifestations of circadian rhythm disruption, this model was producing a dissociation or desynchrony among different circadian rhythms.

With the exception of quiet wakefulness, our finding of a period of approximately 24 h in all output measures suggests that there was no internal desynchrony occurring. T21 cycles with similar and shorter photoperiods to the one used in the present study tend to favor a single (Stephan, 1983), or at least a very strong (Campuzano, 1998) free-running component. It has also been suggested that the presence of wheels can favor the observation of a single free-running rhythm (Campuzano, 1998). The exposure to the T21 cycle was very brief compared to most other studies, so it is possible that misalignment among rhythms (Campuzano, 1998; Cambras et al., 2004, 2007; Vivanco et al., 2010) could develop over time. To our knowledge, sleep in rats has never been assessed in a T21 cycle. It remains possible that as with activity, sleep might only favor a single free-running component in a T21. The data from the present study suggest that misalignment among endogenous oscillators involved in the sleep-wake cycle, likely does not explain the learning and memory impairment elicited by this paradigm.

### Could Circadian Rhythm Misalignment of This Nature Affect Learning and Memory?

At first glance free-running in a light dark cycle appears mundane as endogenous period is maintained. It does, however, mean that processes modulated in part by circadian rhythms, such as activity, feeding, and sleeping are now occurring in the opposite phase of the light dark cycle. Circadian misalignment can mean different things, such as misalignment between different endogenous oscillations (splitting mentioned above), or as in this case misalignment of circadian rhythms with the zeitgebers in the environment (Baron and Reid, 2014). This sort of circadian rhythm misalignment occurs during shift work, and jet lag, as circadian rhythms are maintained but the rhythms are not entrained to the external cycle (Escobar et al., 2011). Perhaps the most apt example of the T21 manipulation in the real-world is N24HSWD. Similar to our model, in N24HSWD tau (endogenous day length) is slower than the T (external day length). In our case approximately 4 h slower which is substantially more than in N24HSWD (approximately 1 h). The mismatch between tau and T displayed in N24HSWD creates a continual state of circadian rhythm misalignment (Baron and Reid, 2014). Circadian misalignment has been associated with increased mortality rate, cancer, depression, metabolic syndrome, cognitive impairments, and obesity (Escobar et al., 2011; Park et al., 2012; Zelinski et al., 2014a).

The finding that the temporal distribution of quiet wakefulness, sleep, and activity changes during T21 exposure are consistent with other studies in rodents that challenge the circadian system with phase advances (Sei et al., 1992; Altimus et al., 2008; Craig and McDonald, 2008; Castanon-Cervantes et al., 2010; Gibson et al., 2010; Loh et al., 2010; Karatsoreos et al., 2011; Legates et al., 2012; Van Dycke et al., 2015), forced activity during the light period (Salgado-Delgado et al., 2008, 2010a,b), or feeding that is restricted to the light phase (Loh et al., 2015). Memory impairments are often found in these models as well (Gibson et al., 2010; Loh et al., 2010, 2015; Karatsoreos et al., 2011; Legates et al., 2012). Exposure to light and dark at times that conflict with the phase of subjective circadian time has harmful effects on areas of the brain involved in learning and memory. Hippocampal clock gene expression, CA1 pyramidal cell dendritic spine density, and neurogenesis are impacted by manipulations such as constant darkness or dim-light at night that utilize phase mismatches between circadian and zeitgeber time (Bedrosian et al., 2011, 2013; Monje et al., 2011). Ipso facto, animals exposed to dim light at night, and constant darkness are impaired in hippocampal dependent tasks (Fonken et al., 2012; Jameie et al., 2016). It remains to be seen if processes underlying learning and memory such as long term potentiation are affected by free-running in an unentrainable light dark cycle. The evidence mentioned above suggests that this could be possible.

### K-Complexes and Sleep Spindles During Circadian Misalignment

To our knowledge no study has extended the findings of sleep assessments in unentrainable light dark cycles to K-complexes and sleep spindles in the rat, which are thought to be important for memory consolidation (Diekelmann and Born, 2010). As with SWS, during T21 exposure these processes were expressed more during lights off than they were in a T24. We did not find any changes in the amount of K-complexes and sleep spindles during T21 exposure. While the medial prefrontal cortex is important for memory consolidation (Euston et al., 2007), the observed stability of sleep spindles and K-complexes suggest that the population neural activities in the rat medial prefrontal cortex from which we recorded from, are resistant to the T21 investigated here.

It was possible that the T21 disrupted the temporal relationship between the cortex and hippocampus, which could elicit a memory deficit even if the features of the cortex alone (e.g., K-complexes and spindles) were not affected. However, we found that the interaction between the cortex and hippocampus, as delineated by K-complex triggered average of the hippocampal LFP, was unaffected by T21 exposure. These data suggest that desynchrony of cortical and hippocampal events are less likely to be responsible for the memory impairment elicited by circadian misalignment. However, it should be noted that we were unable to detect high frequency events, such as SWRs, reliably. It is possible that brain swelling after surgical implantation of electrodes, assaults to the head cap, combined with the maiden use of this wireless recording setup could have contributed to this. Hippocampal differential recordings with the relatively small tip separation (0.4 mm) may have attenuated high frequency signals more strongly because they tend to be local. It is still possible that the temporal relationship between the hippocampal SWRs and the cortical K-complexes and sleep spindles are affected by T21 exposure. Another possibility is that the change in the interval between learning and sleep that occurs during circadian misalignment is affecting consolidation of that information (Diekelmann and Born, 2010). Future studies are needed to determine if sleep that is out of phase with the light-dark cycle specifically affects memory consolidation.

### Limitations

The small sample size in the current study should be taken into account when interpreting the results from the present study. The technical difficulties associated with recording continuously from freely moving animals housed in wheels, resulted in our sample size being smaller than desired. Nonetheless, given that we are recording data continuously in the scale of milliseconds from the same animals over 18 days, for this type of endeavor, we argue that statistical power is adequate. To evaluate the effects of sample size on the observed effects, we can qualitatively compare these data to past data that we have collected. While this is not possible for the sleep data because we have never evaluated it in the context of the T21 before, our running wheel data in five animals is analogous to past reports with bigger sample sizes (Devan et al., 2001; Craig and McDonald, 2008; Zelinski et al., 2013, 2014b; Deibel et al., 2019).

Another factor that must be considered when interpreting the results is that in our T21 paradigm, photoperiod is also manipulated. As a result, it could be contributing to our past and present observations. We have observed memory impairments with a 12L:9D T21 (Devan et al., 2001; Zelinski et al., 2013, 2014b) and as used here, a 9L:12D T21 (Craig and McDonald, 2008). Importantly though we have never tested the 9L:12D T21 iteration in a 6-day paradigm expected to impair memory. The one time we used 6 days of 9L:12D T21, it was followed by 10 days of a normal 12L:12D light dark cycle and then distributed MWT training. As a brief amount of the manipulation was used, followed by 10 days to re-entrain before MWT training began, as expected memory was not impaired. We have not tested a 12L:9D T21 in this manner, but we would also expect to not observe a memory impairment.

Rather than photoperiod, it is our tenet that the memory impairment is due to experiencing the inability to entrain to a light dark cycle. Rats fail to entrain to T21 schedules and exhibit free-running behavior in a variety of photoperiods: 12L:9D (Devan et al., 2001; Zelinski et al., 2013, 2014b; Deibel et al., 2019); 2L:19D (Stephan, 1983); 9L:12D (current data, Craig and McDonald, 2008); 10.5L:10.5D (Campuzano, 1998). When the failure to entrain occurs for a brief period of time before memory acquisition, memory consolidation is unaffected (Craig and McDonald, 2008), but when it occurs during acquisition (Devan et al., 2001), or in between acquisition and retention testing (Zelinski et al., 2014b; Lewis et al., 2020), consolidation is impacted.

A recent report, in which day length and photoperiod varied day to day for 7 days, speaks to this (Lewis et al., 2020). The male Long-Evans rats failed to entrain to this schedule and free-ran at ∼24.5 h. Crucially, as in Zelinski et al. (2014b), the lighting schedule was introduced in between acquisition and retention testing. In accordance with Zelinski et al. (2014b), these rats also displayed impaired retention in the water task. This is surprising given that the Lewis et al. (2020) manipulation is very different as period and photoperiod change from day to day. Importantly though, in both paradigms animals free-run in a light dark cycle that they cannot entrain to. If these observations were relying on photoperiod, one would not expect similar effects on memory between the two manipulations. Further, it is important to note that we have found that a 9L:12D T21 can impair memory, even before learning occurred, albeit with a lot a T21 exposure (Craig and McDonald, 2008). It appears that the inability to entrain to a light dark cycle is deleterious to memory. That being said, photoperiod is involved in this relationship and elucidation of its role is needed to fully understand how circadian rhythms interact with memory processes.

## Conclusion

Six days of T21 exposure had no impact on sleep architecture, including aspects thought to be involved in memory consolidation. The T21 was outside the range of entrainment for both sleep and activity. This means that processes modulated in part by circadian rhythms such as, eating, sleeping, and activity are occurring in the phase of the light dark cycle in which they normally do not occur. The data from the current study indicate that changes in sleep likely are not crucial to the memory impairment elicited by this paradigm. It remains possible that experiencing light or dark at times that conflict with the phase of circadian time during T21 exposure attenuates LTP. But these data suggest that changes in sleep architecture are not responsible for this suspected reduction in plasticity. Temporal redistribution of circadian processes in a light dark cycle occurs in common forms of circadian misalignment. More studies are required to determine the physiological effects of experiencing light or dark when the brain expects the opposite.

## Data Availability Statement

The raw data supporting the conclusions of this article will be made available by the authors, without undue reservation.

## Ethics Statement

All procedures were conducted in accordance with the Canadian Council for Animal Care and approved by the University of Lethbridge Animal Welfare Committee.

## Author Contributions

SD conducted the circadian rhythm analyses and wrote final draft of manuscript. RR was involved in data collection, writing, and electrophysiology data analysis. HS was involved in data collection and editing. KA was involved in electrophysiology data analyses and editing. BM was involved in the editing process. MT was involved in study design, data analysis, and writing. RM was involved in study design and the editing process. All authors contributed to the article and approved the submitted version.

## Conflict of Interest

HS was employed by the company NeuroTek Innovative Technology Inc. The remaining authors declare that the research was conducted in the absence of any commercial or financial relationships that could be construed as a potential conflict of interest.
