# Supplementary material for: Assessment of Sleep, K-Complexes, and Sleep Spindles in a T21 Light-Dark Cycle
Source: Front Neurosci. 2020 Oct 6;14:551843. doi: 10.3389/fnins.2020.551843 (PMC7573124; doi:10.3389/fnins.2020.551843)
Supplement: Supplementary file 1 [file Table_1.DOCX]

A


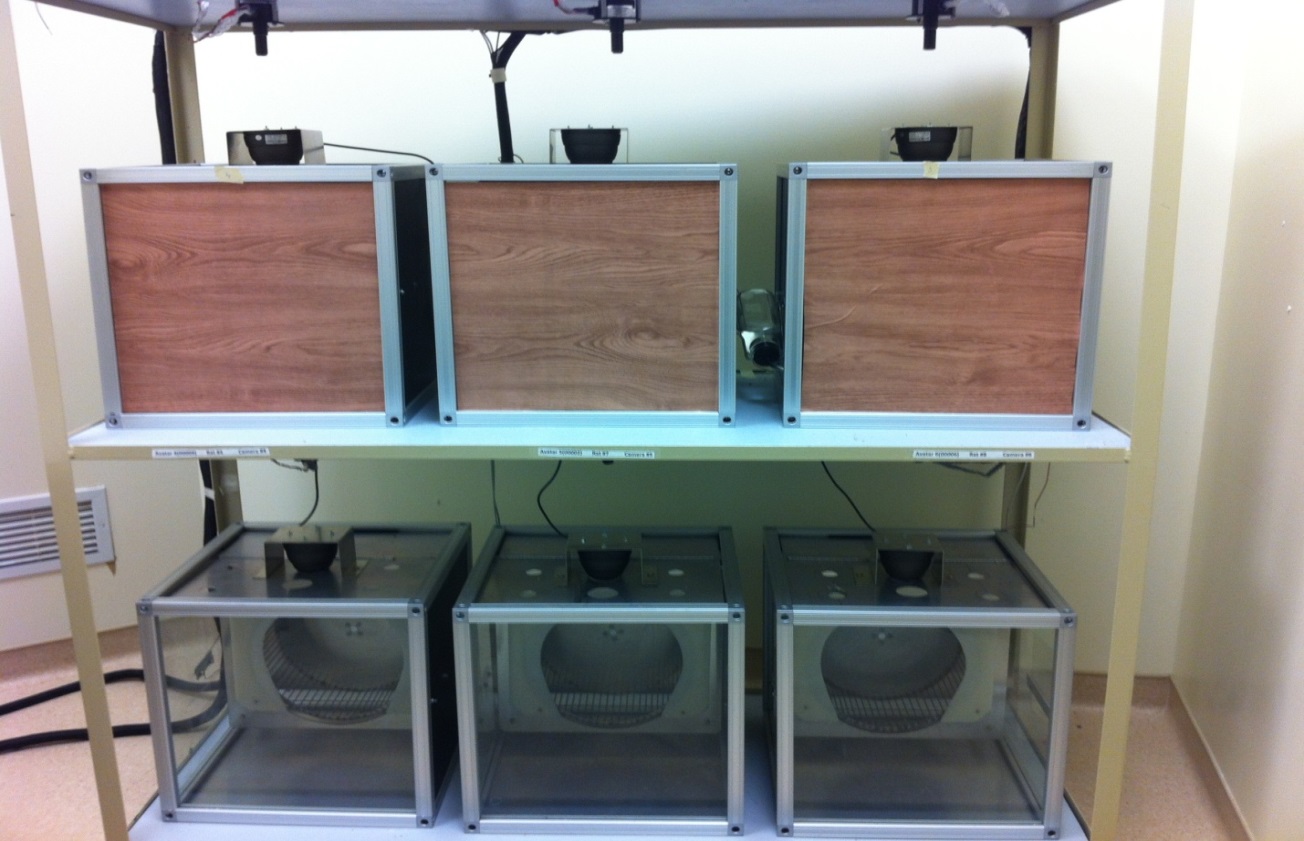


B


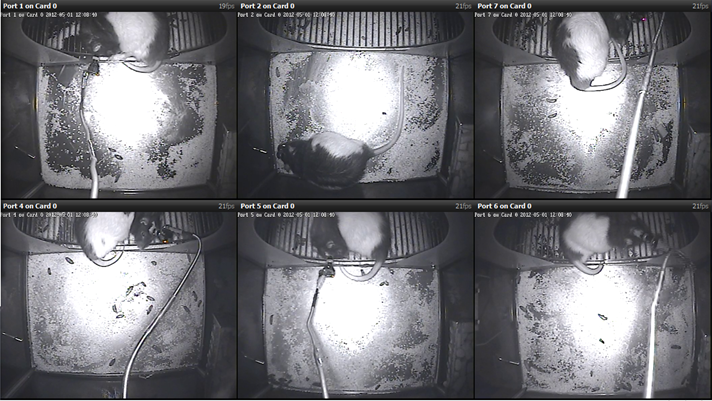


Supplementary Figure 1. Housing units. A) Housing units were designed to provide low noise continuous recording of electrophysiological signals from freely behaving rats. B) Rats were allowed to continuously live in a recording environment for the entire period of the experiment.


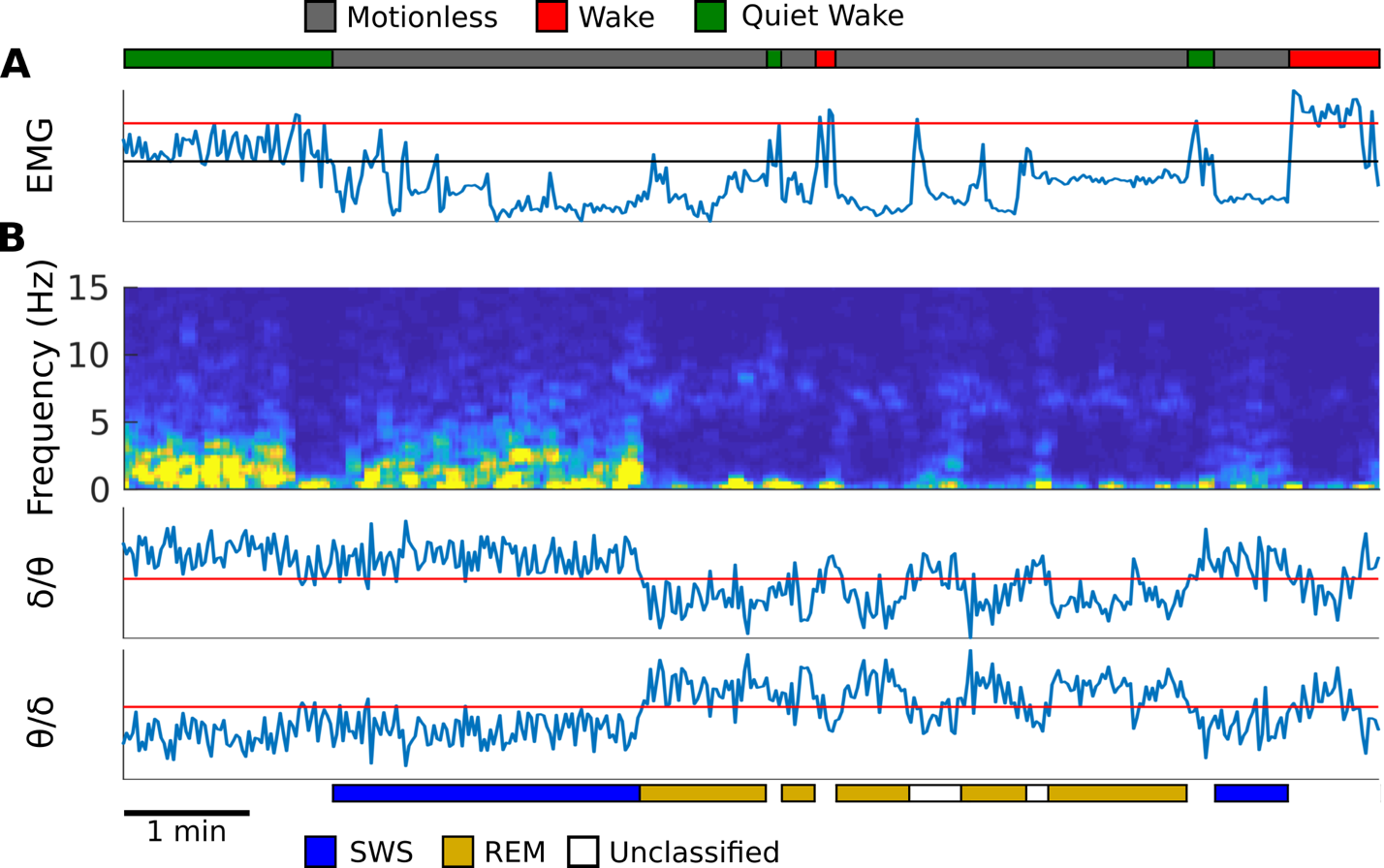


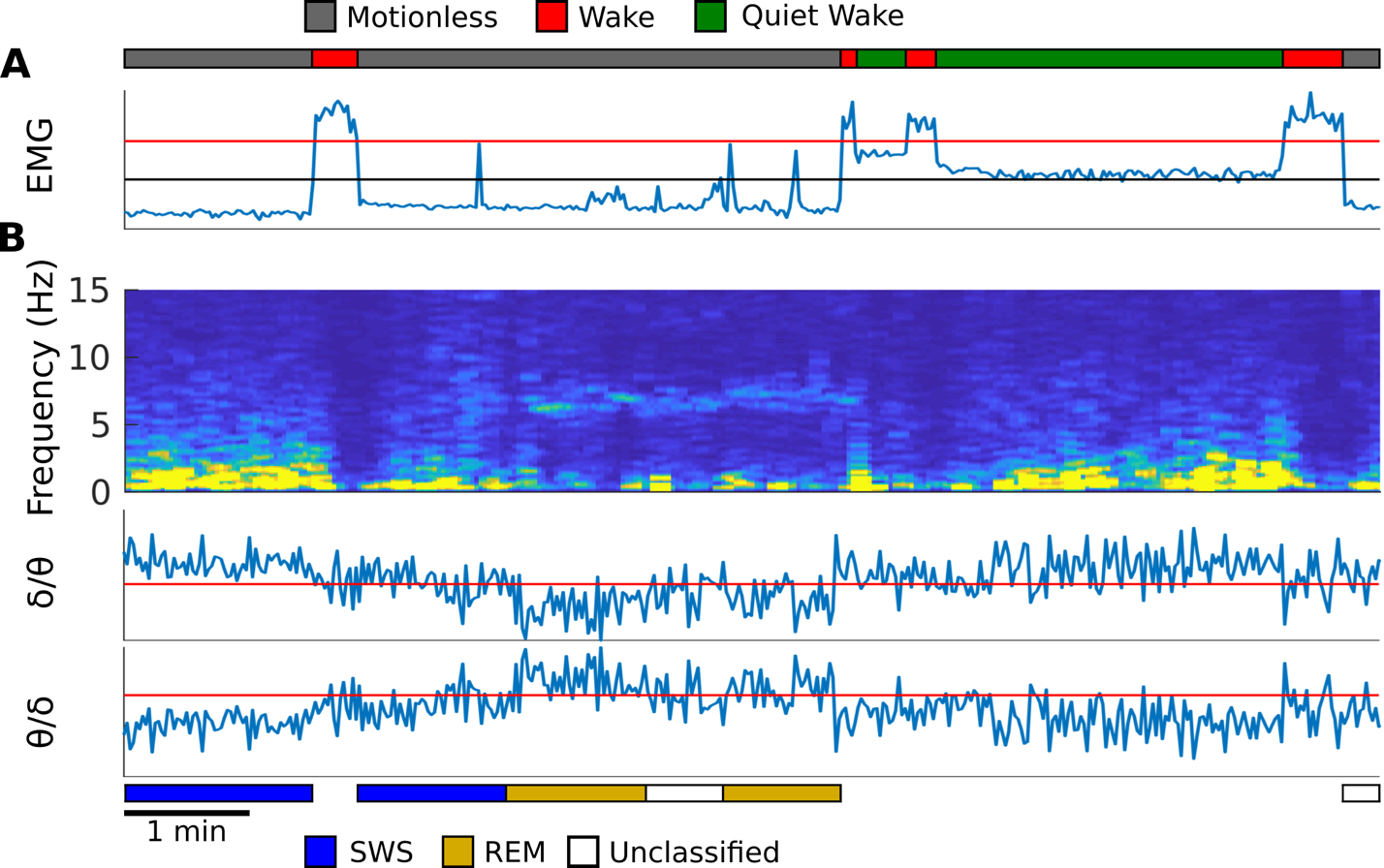


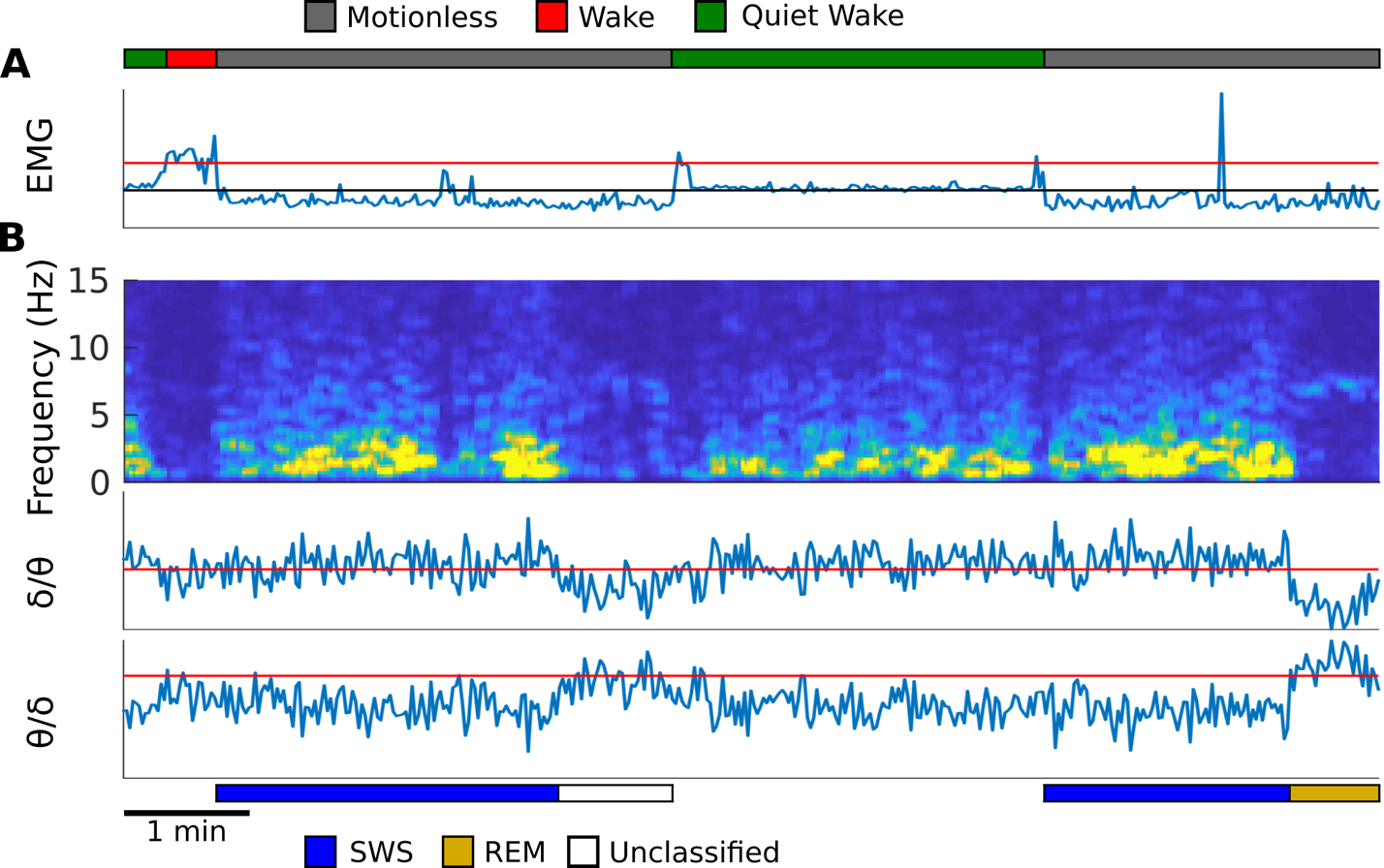


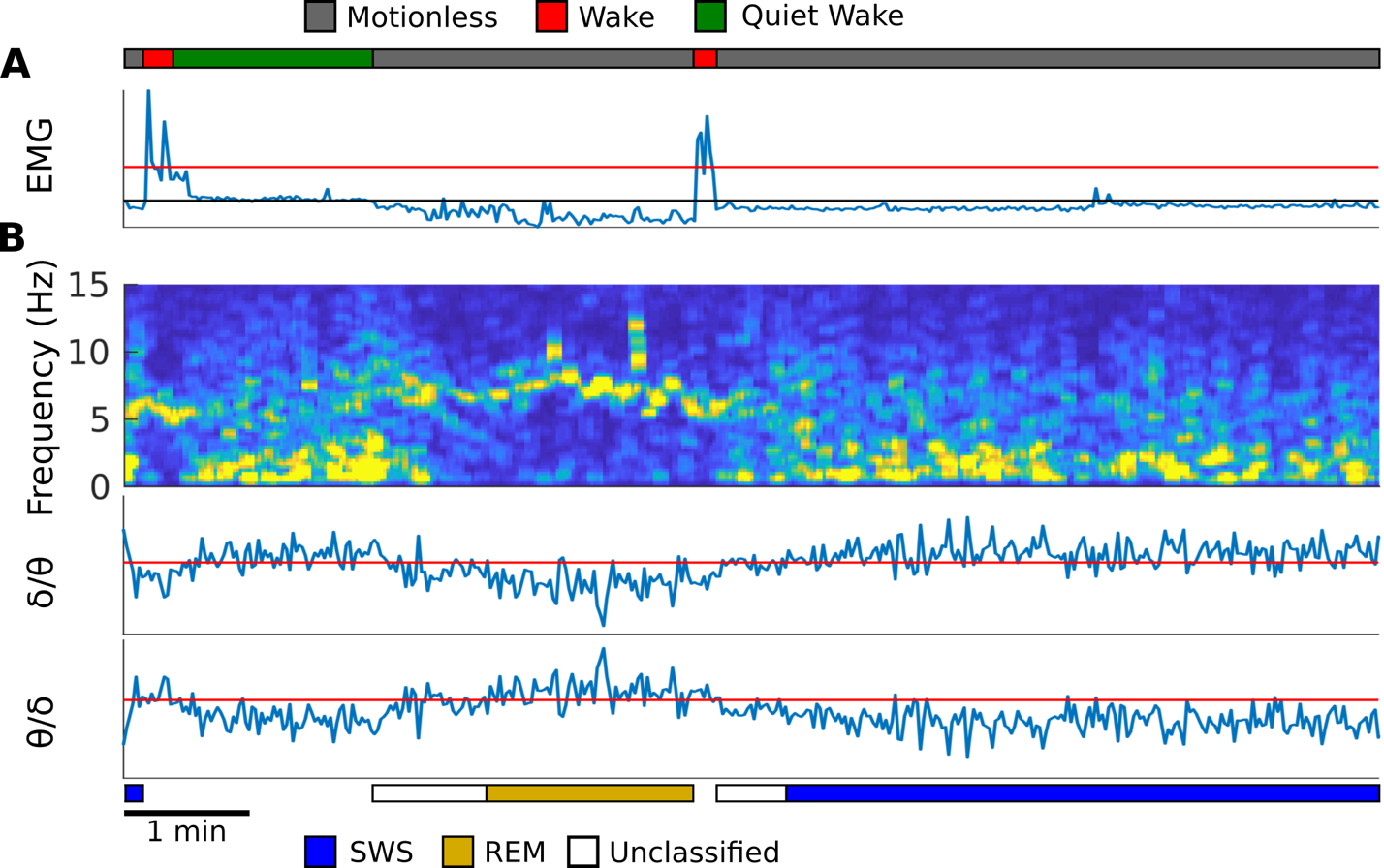


Supplementary Figure 2. Examples of automated sleep state detection for the remaining four animals (one rat is represented in each panel). (A) EMG from the neck muscle (blue trace) was used to score periods of motionless, wake and quiet wake, which were represented as gray, red and green periods in the top trace. EMG power is shown, and the y-axis is in log scale. Red threshold was used to find periods of low EMG power, and the black threshold was used to separate quiet wake from motionless periods. (B) A spectrogram was created from the hippocampal LFP (first panel). The average spectral power for delta (1 – 4Hz) and theta (5 – 10Hz) frequency bands was calculated and the theta to delta ratio (second) and the delta to theta ratio (third) were computed. Within motionless periods, these two ratios were used to identify periods of REM (dark yellow) and SWS (blue), respectively. The periods that did not exceed the thresholds was labeled as unclassified (white). All thresholds were determined so that the agreement between manual scoring and automated scoring was maximized. Temporal resolution for each plot is 2 seconds.


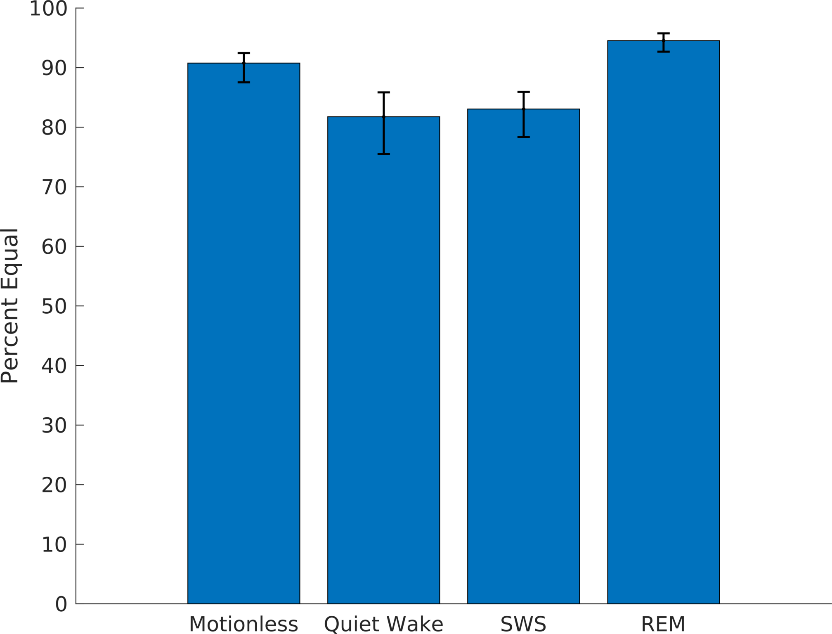
A


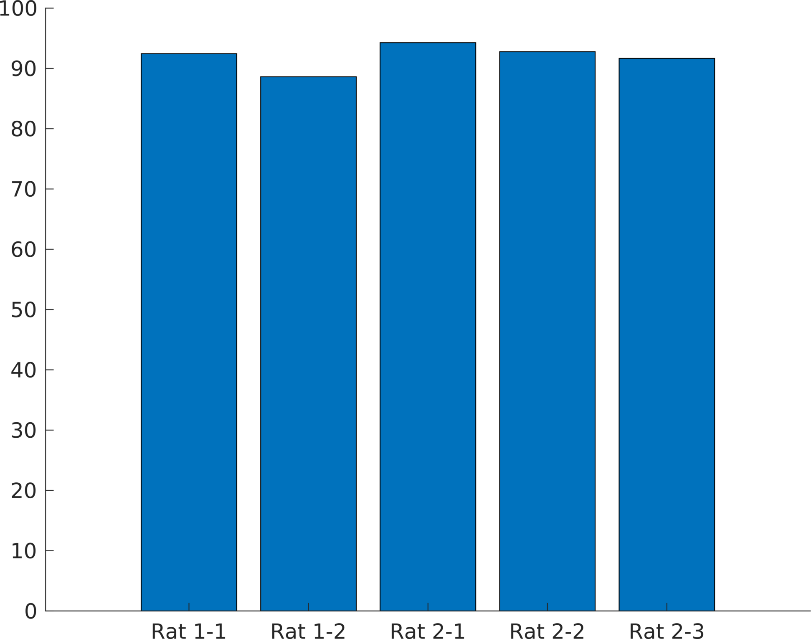


B


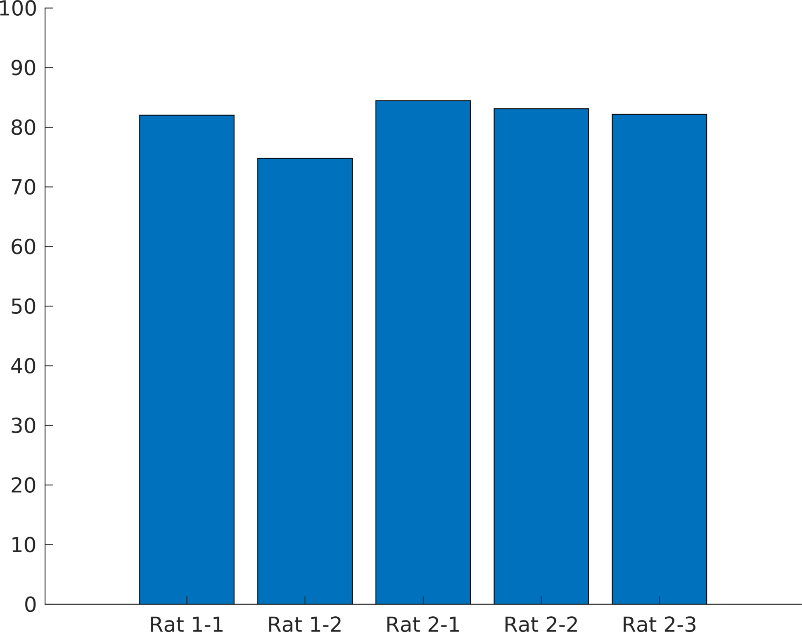


C

Supplementary Figure 3. Percentage of 2-second bins equal between automated and manual scoring for A) Sleep States, B) K-Complexes, and C) Spindles. A) is averaged over days and 5 animals. Error bars represent SEM. B) and C) are one day scored per rat. Due to inconsistent EEG quality over the duration of the experiment only five rats were used

Supplementary Figure 4. Double plotted scale actograms with 10-minute bins per animal for each circadian output variable. Each animal is in the same relative position in all figures. The shaded area represents lights off. A) Activity. B) QW. C) Motionless. D) SWS. E.) REM sleep. F) K-complexes. G) Sleep spindles.
